# Imaging features of extraaxial musculoskeletal tuberculosis

**DOI:** 10.4103/0971-3026.54873

**Published:** 2009-08

**Authors:** Filip M Vanhoenacker, Darshana A Sanghvi, Adelard I De Backer

**Affiliations:** 1 Department of Radiology, Antwerp University Hospital, UZA, University of Antwerp, Wilrijkstraat 10, B-2650 Edegem, Belgium and Department of Radiology, AZ Sint-Maarten, Duffel-Mechelen, Rooienberg 25, B-2570 Duffel, Belgium; 2 Department of Radiology, KEM Hospital and Seth GS Medical College, Acharya Dhonde Marg, Parel, Mumbai, India; 3 Department of Radiology, AZ Sint-Lucas, Groenebriel, 1, B-9000 Ghent, Belgium

**Keywords:** Computed tomography, Magnetic Resonance Imaging, musculoskeletal, radiography, tuberculosis, ultrasound

## Abstract

Tuberculosis (TB) continues to be a public health problem in both developing and industrialized countries. TB can involve pulmonary as well as extrapulmonary sites. The musculoskeletal system is involved in 1–3% of patients with tuberculosis. Although musculoskeletal TB has become uncommon in the Western world, it remains a huge problem in Asia, Africa, and many developing countries. Tuberculous spondylitis is the most common form of musculoskeletal TB and accounts for approximately 50% of cases. Extraspinal musculoskeletal TB shows a predilection for large joints (hip and knee) and para-articular areas; isolated soft tissue TB is extremely rare. Early diagnosis and prompt treatment are mandatory to prevent serious destruction of joints and skeletal deformity. However, due to the nonspecific and often indolent clinical presentation, the diagnosis may be delayed. Radiological assessment is often the first step in the diagnostic workup of patients with musculoskeletal TB and further investigations are decided by the findings on radiography. Both the radiologist and the clinician should be aware of the possibility of this diagnosis. In this manuscript we review the imaging features of extraspinal bone, joint, and soft tissue TB.

## Introduction

Tuberculosis (TB) is still a major public health problem in both developing and industrialized countries. It is endemic in most of the developing countries. Although the incidence of TB has decreased since the introduction of antituberculous drugs, most developing countries have been facing a resurgence of the disease since 1985. Several factors contribute to the increase in the incidence of TB in the developed world; these include immigration from countries with high prevalence; a growing elderly population debilitated with other diseases (e.g., diabetes mellitus, chronic renal failure, chronic obstructive disease, liver cirrhosis, lymphoproliferative disorders, etc.); a growing number of immunocompromised patients; the emergence of multidrug-resistant TB; and various socioeconomic factors (e.g., alcohol and drug abuse, poverty, homelessness, etc.). The prevalence of TB is particularly high among patients with AIDS, and the disease is often the first manifestation of HIV infection.[1–3]

Extrapulmonary manifestations are estimated to occur in approximately 20% of patients with TB.[4] Musculoskeletal TB accounts for 1–3% of tuberculous infections. The most common form of musculoskeletal TB is tuberculous spondylitis (50%). Extraspinal manifestations are the least common[5]; the reported frequency of peripheral arthritis is 60%, of osteomyelitis 38%, and of tenosynovitis and bursitis 2%.[6–9] Intercurrent active pulmonary TB is only seen in about one half of the patients.[3]

**Figure 1 (A,B):**
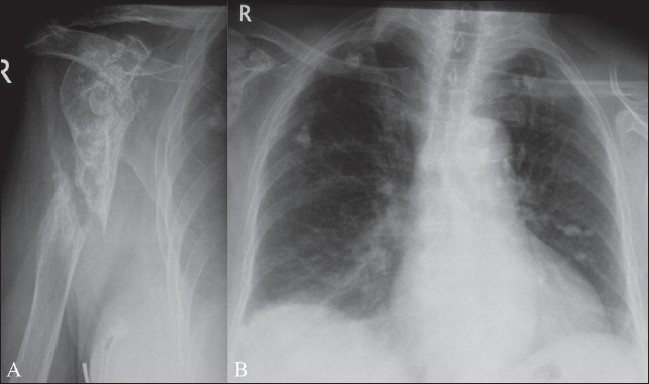
Pseudarthrosis of the humerus, as a complication of chronic tuberculous osteomyelitis at the site of a fracture. Plain radiograph of the right humerus (A) shows irregular sclerosis of the proximal humeral diaphysis and pseudarthrosis of the diaphysis. Note also partial destruction of the humeral head due to sequelae of associated tuberculous arthritis of the shoulder. Plain radiograph (B) of the chest reveals bilateral calcified granulomas within the lungs

The nonspecific, often indolent, clinical presentation of extraspinal musculoskeletal TB, together with its low prevalence and the low index of suspicion among clinicians, may result in delay in the diagnosis. However, prompt diagnosis and treatment of this curable disease remains critical for planning proper management and preventing joint deformity and permanent bone destruction [Figure 1].[10] Although diagnosis of extraspinal musculoskeletal TB is not possible solely on the basis of the clinical or imaging findings, imaging may guide further diagnostic workup and may result in earlier and adequate treatment. Histopathological examinations, culture identification, and polymerase chain reaction (PCR) are among the most accurate methods for diagnosis.

The purpose of this paper is to review the imaging features of extraspinal musculoskeletal TB. The merits of each imaging modality will be emphasized.

## Pathogenesis of extraspinal musculoskeletal TB

### Causative organisms

*Mycobacterium tuberculosis* is the main causative organism and only a few cases are attributable to *Mycobacterium bovis*. Atypical mycobacteria, such as *Mycobacterium kansasii, Mycobacterium marinum, Mycobacterium scrofulaceum*, and *Mycobacterium avium* complex account for approximately 1–4% of cases of TB.[11]

### General mechanisms of spread

Musculoskeletal TB mostly results from hematogenous dissemination of mycobacteria or lymphogenous spread from a primary or reactivated focus of infection.[12] Rarely, musculoskeletal TB may be the result of direct inoculation of the organism into the site.[13–15] Furthermore, injuries may also result in reactivation of dormant infection.[16]

### TB of joints

TB of a joint may result from hematogenous dissemination through the subsynovial vessels or, indirectly, from epiphyseal (more common in adults) or metaphyseal (more common in children) lesions that erode into the joint space. Transphyseal spread, typical of TB, is very unusual in pyogenic arthritis.[17]

Reactive hyperemia results in marked juxta-articular bone demineralization and local bone destruction. Later in the disease, periosteal new bone formation may occur. When the disease process reaches the subchondral region, the articular cartilage loses its nutrition and gets detached from the bone; this may result in loose bodies of cartilage within the joint. Damage to the physis in childhood may result in shortening or angulation of the limb.

When the infection starts as a synovitis, the synovial membrane becomes congested and thickened and joint effusion develops. Granulomatous synovial lesions expand over the bone at the synovial reflections, with subsequent development of erosions and cartilage destruction. However, because the exudate in tuberculous arthritis lacks proteolytic enzymes, cartilage loss occurs only late in the disease.[18] With further disease progression, osteolytic bone lesions develop.[11] In long-standing disease, flakes or loose sheets of necrotic articular cartilage and accumulations of fibrinous material in the synovial fluid may produce the rice bodies found in synovial joints, tendon sheaths, and bursae. When untreated, progression of tuberculous arthritis may result in para-articular soft tissue masses and cold abscesses, and sinus tracts may develop.[18]

Poncet's arthritis has been traditionally defined as a specific noninfectious form of tuberculous rheumatism with polyarticular involvement in patients with active or inactive visceral TB. Its existence as a distinct entity is, however, still a matter of debate.

### Tuberculous osteomyelitis

The bones may be involved as a result of hematogenous spread from a primary focus, which is usually in the lung or the lymphatic system. In tuberculous osteomyelitis, a granulomatous lesion develops within the bone at the site of deposition of the mycobacterium, usually the metaphysis. As the infected focus enlarges, caseation and liquefaction necrosis occur, with resorption of bone trabeculae. Further disease progression may result in macroscopically visible bone destruction, transphyseal spread of disease, and joint involvement.[18] Rarely, lesions involve the diaphysis; cortical destruction may then occur, with subsequent development of a periosteal reaction and a paraosseous soft tissue mass or collection.[17] Multifocal bone involvement may be seen, with the lesions at different stages of development; this is due to hematogenous spread, with bacilli being seeded at different times. A suppressed host immune response predisposes to multiple bone lesions.

### Tuberculous tenosynovitis and bursitis

Tuberculous tenosynovitis may result from hematogenous spread or it may be due to periarticular extension of tuberculous arthritis. Either tendon or synovium, or both, may be infiltrated and thickened. Tubercle formation may result in caseation, necrosis and secondary effusion within the tendon sheath. Disease progression may lead to thinning of the tendon and tendon rupture. Sinus formation is rare.

Secondary tuberculous involvement of the synovium of bursae is well known, but primary bursitis due to hematogenous spread is rare.[7]

### Tuberculous myositis

Skeletal muscle involvement without bony involvement, resulting from hematogenous or lymphatic spread, is extremely rare. In most cases, tuberculous involvement of the muscles results from disease progression and local spread from adjacent tuberculous lymphadenitis, osteomyelitis, or arthritis. Direct inoculation by syringes contaminated with *M tuberculosis* is another possible pathogenetic mechanism.[14] The lesions tend to spread along the route offering least resistance and thus track along the fascial planes.[19]

## Imaging

### Joint TB

**Figure 2 (A-D):**
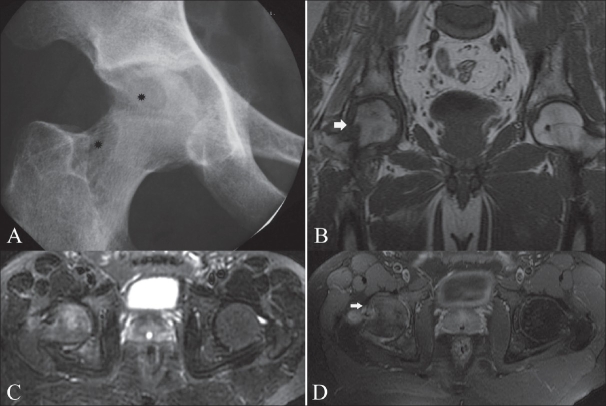
Tuberculous arthritis of the right hip. Plain radiograph (A) of the right hip demonstrates periarticular osteopenia, indistinct margins of the articular surfaces, and erosions of the femoral head and neck (asterisks). Coronal T1W MRI (B) shows a large erosion in the femoral neck (white arrow) and hypointense bone marrow edema within the acetabulum and lateral aspect of the femoral head. Axial fat-suppressed T2W MRI (C) shows hyperintense bone marrow edema in the right femoral head and acetabulum. Axial fat-suppressed T1W MRI (D) after gadolinium contrast administration shows peripheral enhancement of the large erosion in the femoral neck (white arrow). Note also some enhancement of the bone marrow of the femoral head and acetabulum as well as of the joint space

**Figure 3:**
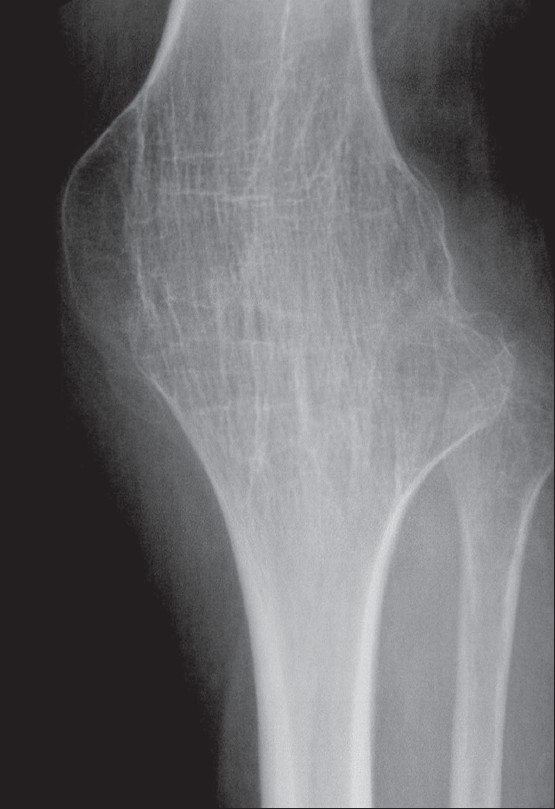
Plain radiograph shows bony ankylosis of the left knee, secondary to prior surgical intervention for end-stage destructive tuberculous arthropathy

**Figure 4 (A-E):**
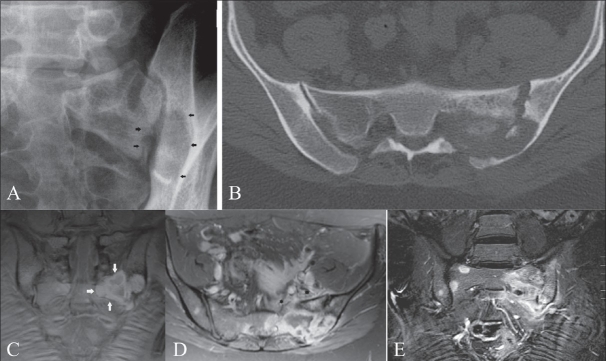
Tuberculous sacroiliac joint infection. Plain film of the left sacroiliac joint (A) shows large erosions with surrounding sclerosis on both sides of the left sacroiliac joint (black arrows). CT scan of the sacroiliac joints (B) confirms marked destruction of the left sacroiliac joint. Coronal fat-suppressed T1W MRI (C) shows a hyperintense peripheral rim around an intraosseous abscess within the left sacrum (arrows). Axial, contrast-enhanced, fat-suppressed T1W MRI (D) shows peripheral enhancement of the large subchondral erosions and adjacent intraosseous abscess. Note the enhancement of the synovium and of the soft tissues anterior to the left sacroiliac joint (asterisks). Coronal, contrast-enhanced, fat-suppressed T1W MRI (E) shows contrast uptake on both sides of the left sacroiliac joint, absence of contrast uptake within the central part of joint and multiple areas of contrast uptake within the sacrum and spine, due to associated disseminated tuberculous osteomyelitis

**Figure 5 (A-D):**
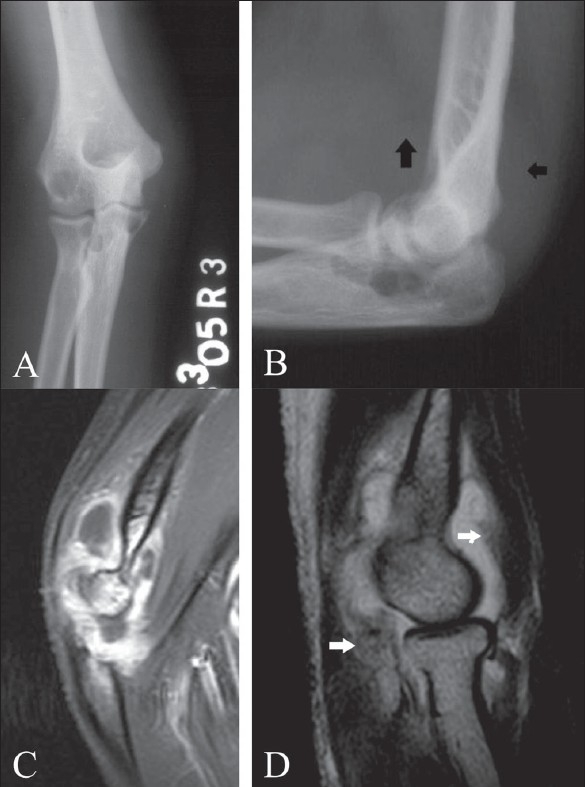
Tuberculous arthritis of the right elbow. Frontal (A) and lateral (B) plain radiographs demonstrate multiple osteolytic lesions within the ulna, radius and radial condyle. Note also joint effusion with displacement of the peri-articular fat pads of the elbow (arrows). Sagittal, contrast-enhanced, fat-suppressed T1W MRI (C) shows peripheral enhancement of the synovium. Note also enhancement of the adjacent bone marrow. Sagittal, fat-suppressed T2W MRI (D) in another patient shows joint effusion and a relatively hypointense signal of the thickened synovial tissue (white arrows)

**Figure 6:**
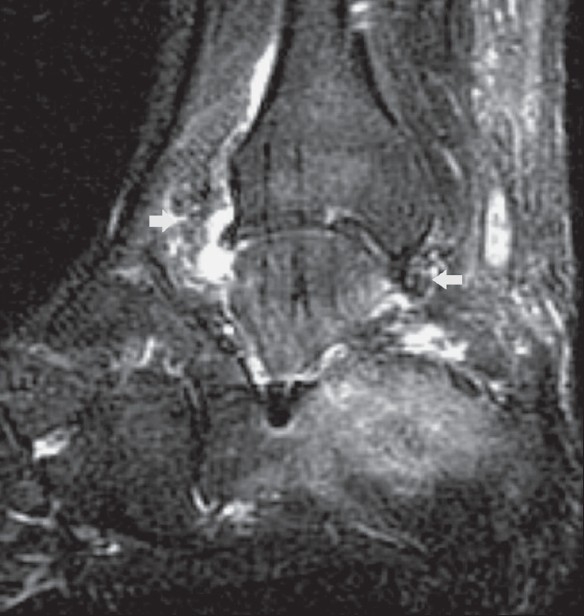
Tuberculous arthritis of the right ankle joint. Sagittal, fatsuppressed T2W MRI shows hyperintense joint effusion within the tibiotalar and subtalar joints. The thickened synovium is hypointense (white arrows). Note also bone marrow edema in the distal tibia, talus and calcaneum

**Figure 7:**
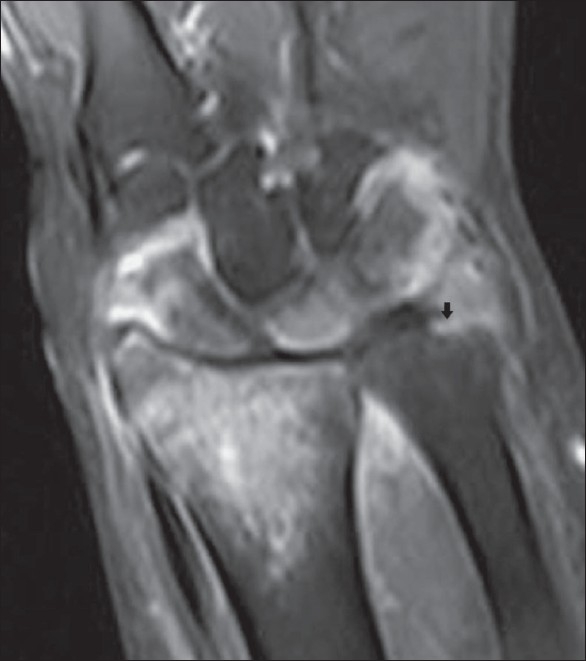
Tuberculous arthritis of the wrist. Coronal, fat-suppressed T2W MRI shows hyperintense bone marrow edema involving the distal radius, scaphoid, lunate and triquetrum. Note increased joint fluid in the distal radioulnar and radiocarpal joints and erosion of the distal ulna (black arrow)

**Figure 8:**
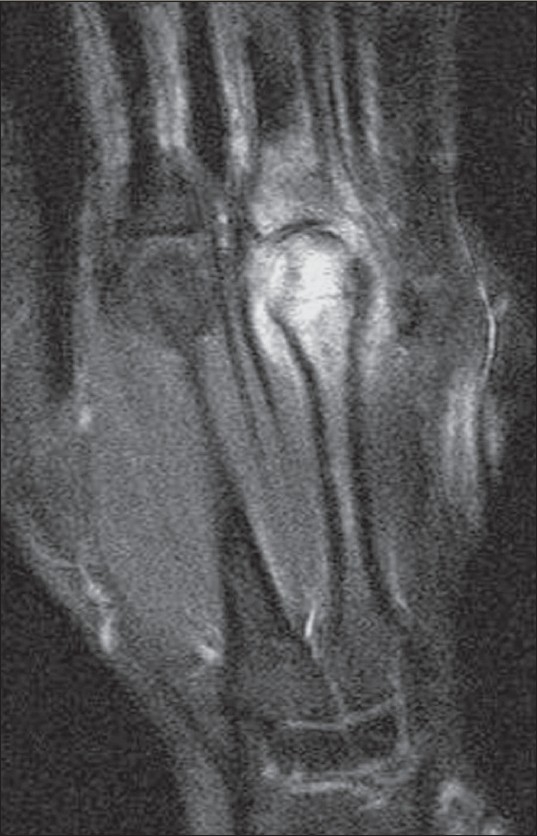
Tuberculous arthritis of the metacarpophalangeal joint. Coronal, fat-suppressed T2W MRI shows hyperintense bone marrow edema on both sides of the third metacarpophalangeal joint with joint fluid effusion. The margin of the subchondral cortical bone of the head of the metacarpal bone is indistinct

*Location*: As with most infectious diseases of joints, tuberculous arthritis is characteristically monoarticular; however, in approximately 10% of patients, multifocal joint disease does occur.[18] The most commonly involved joints are the hip [Figure 2] and knee [Figure 3] followed by, in order of frequency, the sacroiliac [Figure 4], shoulder [Figure 1], elbow [Figure 5], and ankle joints [Figure 6]. Peripheral locations [Figure 7] seem to be more frequent than originally reported.[3] Tuberculous lesions are not uncommonly seen in the small bones of the hands [Figure 8] and feet, especially in children, elderly patients, and immunocompromised patients.

*Plain radiographic findings*: A triad of radiologic abnormalities—periarticular osteoporosis, peripherally located osseous erosion, and gradual diminution of the joint space [Figure 2] (Phemister's triad)—is highly suggestive of TB of a joint. [18–22] Other radiographic features include joint effusion [Figure 5] and osteolytic bone destruction.[17,18] Peripherally located osseous erosions are characteristic features of TB in ‘tight’ or weight-bearing articulations such as the hip [Figure 2], knee, and ankle. Occasionally, wedge-shaped areas of necrosis (kissing sequestra) may be present on both sides of the affected joint. Bone sclerosis and periostitis occur late in the disease, except in children, in whom a layered periosteal reaction may be seen. There is, however, no single radiographic feature that allows a specific radiologic diagnosis of joint TB. The end stage of tuberculous arthritis is characterized by severe joint destruction and, eventually, sclerosis and fibrous ankylosis when the active infectious stage has slowly extinguished. In contrast to pyogenic arthritis, the development of bone ankylosis is uncommon in tuberculous arthritis and, when present, is more likely to be secondary to prior surgical intervention [Figure 3].

Although conventional radiography is the appropriate initial imaging study for the evaluation of musculoskeletal TB, plain films may be negative early in the disease.

*Ultrasound findings*: USG may demonstrate the presence of joint effusions. It may also be helpful during aspiration of these effusions for microbiological and histopathological examination and PCR. However, USG is not specific in the diagnosis of joint TB.

**Figure 9 (A,B):**
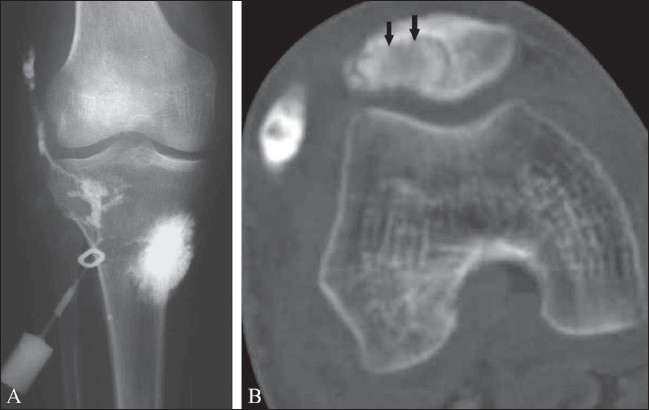
Tuberculous arthritis with fistula formation and chronic osteomyelitis of the patella. Frontal radiograph of the knee after a sinogram (A) shows sinus tract formation. CT scan after a sinogram (B) demonstrates contrast filling of a para-articular collection and a sequestrum within an osteolytic lesion in the patella (black arrows)

*CT Scan*: CT scan is particularly useful for evaluating the degree of bone destruction, sequestrum formation (although rare), and surrounding soft tissue extension [Figure 9].

*MRI*: MRI [Figures 2 and 4–8] is the modality of choice for early detection of joint TB. Synovial proliferation due to tuberculous arthritis is typically hypointense on T2W images [Figures 5 and 6], which may be a very helpful sign for differentiating tuberculous arthritis from other proliferative synovial arthropathies.[23,24] According to Suh *et al*.,[8] this relatively low signal intensity may be due to the presence of hemorrhage, inflammatory debris, fibrosis, and caseation necrosis. After administration of intravenous gadolinium contrast the thickened synovium enhances vividly. Additionally, focal areas of normal-appearing chondral elements may be demonstrated. Chondral lesions and subchondral bone erosions may be visible at a stage when the joint space is still well preserved. Associated bone marrow edema [Figures 2 and 5–8], osteomyelitis, and soft tissue abnormalities such as myositis, cellulitis, para-articular abscess formation, tenosynovitis, bursitis, and skin ulceration/sinus tract formation may be seen.[23] Sinus tracts are characterized by linear high signal intensity on T2W images with marginal ‘tram-track enhancement’ on gadolinium-enhanced images.[25] Para-articular abscesses mostly show a thin and smooth enhancing wall.[24] Definitive diagnosis of TB requires aspiration or synovial biopsy.

### Tuberculous osteomyelitis

*Location*: Virtually any bone may be affected, but tuberculous osteomyelitis occurs most commonly in the bones of the extremities, including the small bones of the hands and feet. The ribs are also frequently involved. Multiple or solitary bone lesions may be seen.[17,26] The metaphysis is the most frequent site of involvement but in rare cases the diaphysis may also be affected.

**Figure 10:**
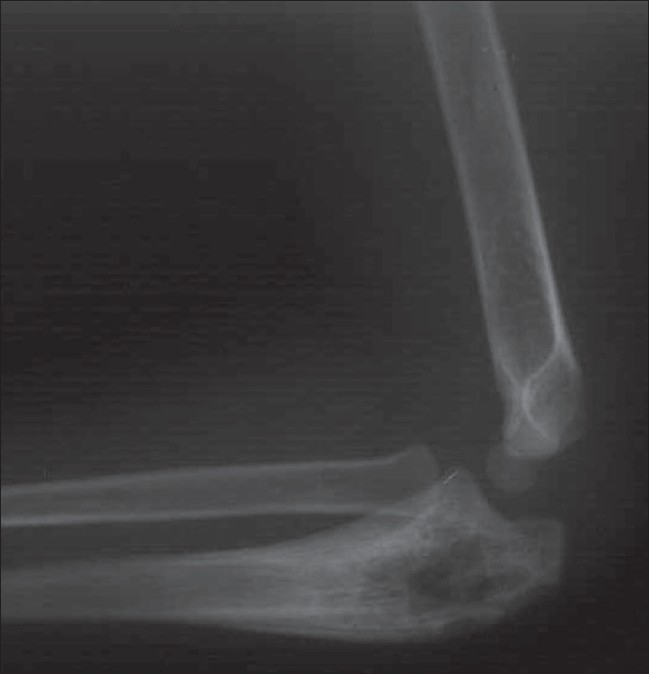
Chronic tuberculous osteomyelitis of the ulna in a child. Plain radiograph of the right forearm shows osteolytic destruction of the proximal ulna with surrounding sclerosis and cortical thickening

**Figure 11 (A,B):**
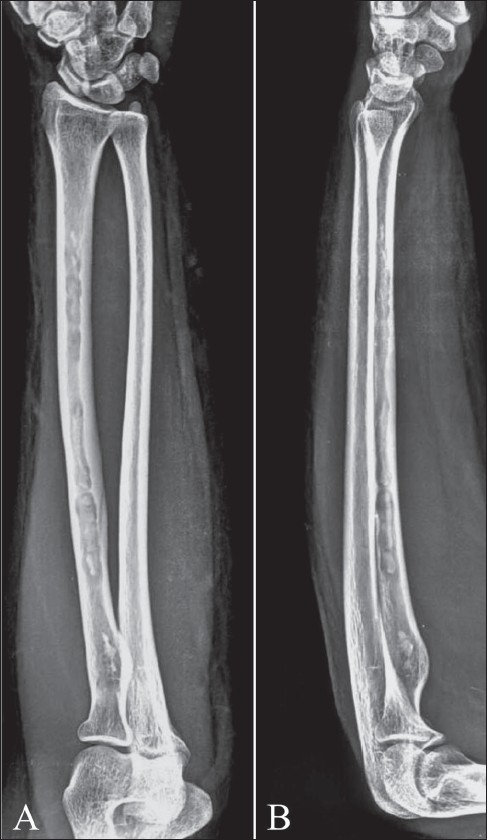
Chronic tuberculous osteomyelitis of the radius in an adult patient. Frontal (A) and lateral (B) radiographs of the left forearm show multiple areas of bone destruction with intralesional sequestration in the diaphysis of the radius. Note also diffuse involucrum formation

*Plain radiographic and CT scan findings*: Plain radiography in osteomyelitis may show soft tissue swelling, minimal periosteal reaction, osteolysis with little or no reactive change, periarticular osteoporosis, and erosions. Sclerosis is less frequently seen [Figure 10]. Sequestration in tuberculous osteomyelitis is relatively uncommon [Figure 11] and less extensive than with pyogenic osteomyelitis.[16,17] CT may be useful for accurate assessment of sequestrum formation [Figure 9].

Multifocal tuberculous osteomyelitis is also known as osteitis cystica tuberculosa multiplex. Multiple sites of involvement are usually seen in children, while in adults, involvement is more often confined to a single bone. The radiographic appearance may be somewhat different in children as compared to adults. In young patients, the lesions usually are osteolytic and well defined, without sclerosis, and may show variable size. Lesion growth may cause metaphyseal expansion. In adults, the lesions are smaller, are located in the long axis of bone, and may show well-defined sclerotic margins.[20,27,28]

**Figure 12 (A,B):**
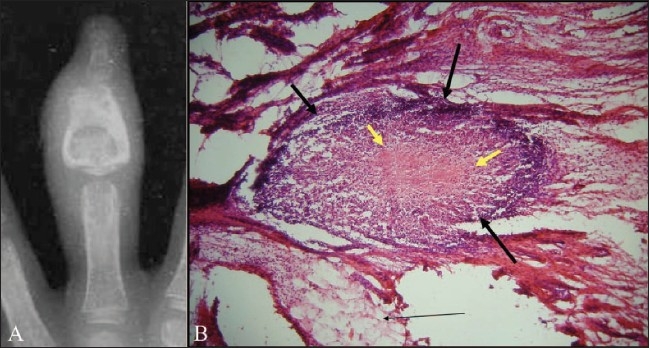
Tuberculous dactylitis in a child: Plain radiograph of the left third finger (A) shows an osteolytic lesion within the middle phalanx of the left third finger. There is fusiform expansion of the bone and the cortex is thickened. Note associated soft tissue swelling. This radiographic appearance is also known as ‘spina ventosa.’ Microphotograph of the histological specimen (B) obtained after synovial biopsy of the adjacent joint shows a caseating epitheloid granuloma (thick black arrows) with central caseating necrosis (thick yellow arrows)

‘Spina ventosa’ (*spina*: a short bone; *ventosa*: inflated with air) is a term used to describe a form of tuberculous osteomyelitis characterized by bone destruction, overlying periosteal thickening, and fusiform expansion of the bone [Figure 12]. Most often, these lesions are seen in the short bones of the hands and feet and are better known as tuberculous dactylitis.[29,30] On imaging, these lesions appear as cyst-like cavities, with expansion of the diaphysis and overlying soft tissue swelling. Bone sequestration may also be present. Multifocal tuberculous dactylitis affecting both hands has been reported recently.[31]

*Ultrasound*: Unless there are associated soft tissue lesions or joint involvement, there is no clear role for USG in the diagnosis of tuberculous osteomyelitis.

**Figure 13 (A,B):**
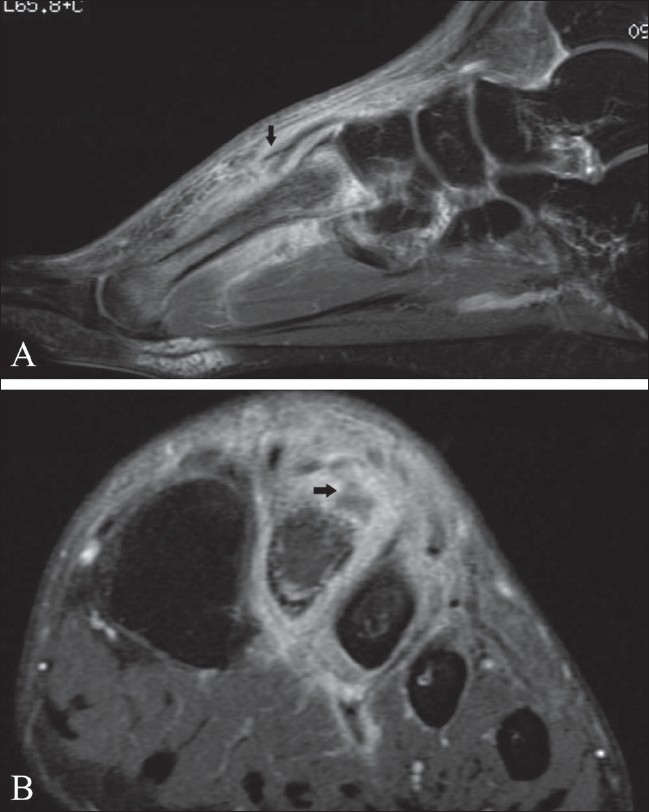
Tuberculous osteomyelitis of the second metatarsal bone of the left foot. Sagittal, fat-suppressed, T2W MRI (A) shows bone marrow edema, adjacent tenosynovitis (arrow) of the extensor tendon and increased signal within the adjacent soft tissues on the plantar and dorsal aspects of the foot. Coronal, contrast-enhanced, fat-suppressed, T1W MRI (B) shows marked enhancement of the soft tissue involvement with central necrosis and abscess formation on the dorsal aspect of the second metatarsal bone (arrow). Note the hazy contour of the dorsal cortex of the diaphysis

*MRI*: MRI may demonstrate intraosseous involvement earlier than the other imaging modalities. Marrow changes are demonstrated as areas of low and high signal intensity on T1W and T2W weighted images [Figure 13], respectively, and show enhancement after intravenous administration of gadolinium chelates. Areas of necrosis appear hyperintense on T2W images and show no enhancement. Deep soft tissue fistulae, sinus tracts, and abscesses are better delineated on gadolinium-enhanced images [Figure 13].

### Tuberculous tenosynovitis

*Location*: Primary tuberculous tenosynovitis is considered an extremely rare condition. It most commonly involves the flexor tendon sheaths of the dominant hand.[6]

*Ultrasound*: USG is the primary investigation to confirm the diagnosis of tenosynovitis and to reveal the degree and extent of tendon and tendon sheath involvement. As in other forms of chronic tenosynovitis, tendon and synovial thickening predominate, with relatively little synovial sheath effusion; in contrast, in acute suppurative tenosynovitis synovial sheath effusion is the predominant feature.

*MRI*: MRI may be helpful to delineate the precise extent of soft tissue involvement and any associated osseous or joint involvement. On MRI, the appearance of tuberculous tenosynovitis depends largely on the duration of the disease. Jaovisidha *et al*.,[6] described three stages of tuberculous tenosynovitis: the hygromatous, serofibrinous, and fungoid stages. The hygromatous stage is characterized by the presence of fluid inside the tendon sheath without associated sheath thickening. The serofibrinous stage is characterized by thickening of the flexor tendons and synovium, with multiple tiny hypointense nodules within the hyperintense synovial fluid on T2W images. These tiny nodules correspond to the rice bodies previously reported in the literature. Finally, there is the fungoid stage, which is characterized by a soft tissue mass involving the tendon and tendon sheath.

### Tuberculous bursitis

**Figure 14:**
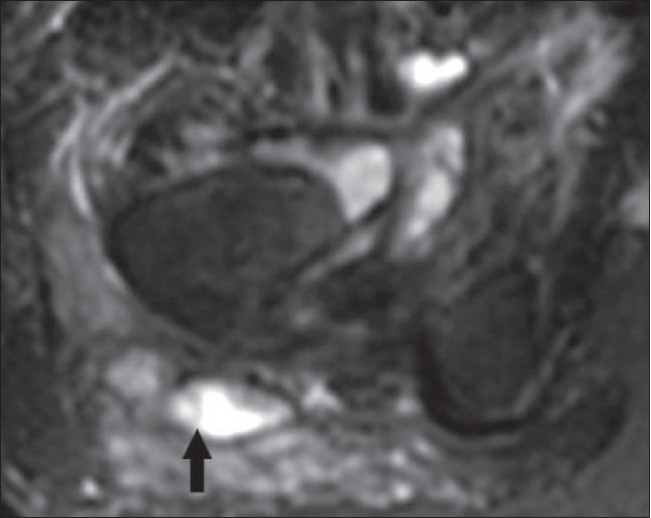
Tuberculous bursitis of the right trochanteric bursa (arrow) on an axial, fat-suppressed T2W MRI. The distended bursa contains fluid, whereas the thickened synovium is of relatively low signal intensity

*Location*: The trochanteric [Figure 14], subacromial, subgluteal, and radioulnar wrist bursae are most commonly affected.[33]

*Plain radiography*: Long-standing bursitis is usually complicated by local osteopenia due to hyperemia, while local pressure of the enlarged bursa may result in focal osteolytic bone destruction (e.g., greater trochanter or the humeral head). The wall of the distended bursa may contain calcifications, which may be visible on radiographs.

*MRI:* Two patterns of involvement have been reported on MRI: a uniform distension of the bursa or multiple small abscesses in the bursa.[34] Low signal intensity material within the fluid-filled bursa on T2W images [Figure 14] is due to the presence of caseous necrosis and fibrotic material.[35]

### Tuberculous myositis

Tuberculous involvement of the muscle or deep fascia is a rare form of musculoskeletal TB and is mostly seen in immunosuppressed patients. Striated muscle is one of the most resistant tissues to mycobacterial infection. This has been attributed to several factors, including poor oxygen content, high lactic acid concentration, and a paucity of reticuloendothelial tissue.[36,37]

**Figure 15:**
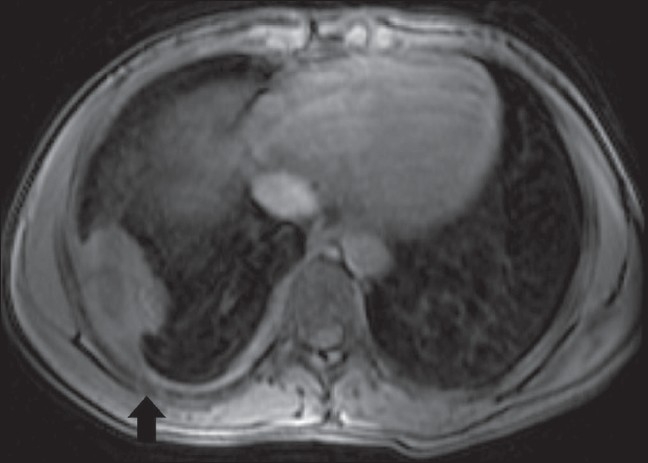
Chest wall tuberculosis. Axial, contrast-enhanced, fatsuppressed, T1W MRI shows a pleural-based mass along the dorsal aspect of the right thoracic wall, with irregular peripheral enhancement. Note the hazy contours of the adjacent rib (arrow) due to osteomyelitis

*Location*: Any muscle may be involved and there are rare case reports of tuberculous pyomyositis affecting the muscles of the upper and lower extremities as well as of the chest and abdominal wall.[20,37–39] Tuberculous granulomatous inflammation and abscesses of the chest wall are most commonly found in the parasternal region, costovertebral junction, and along the shafts of the ribs.[39] There is usually associated tuberculous pleuritis [Figure 15] and intrathoracic lymph node enlargement in contiguity with these chest wall collections. The internal mammary nodes are most commonly involved. It is believed that lymph node enlargement and subsequent caseation necrosis may burrow through the chest wall to form these soft tissue collections.

*Ultrasound*: Tuberculous pyomyositis has been rarely reported on USG.[38] As in tuberculous arthritis, USG may be helpful for guided aspiration of the collections for microbiological or histopathological examination and PCR.[40]

**Figure 16:**
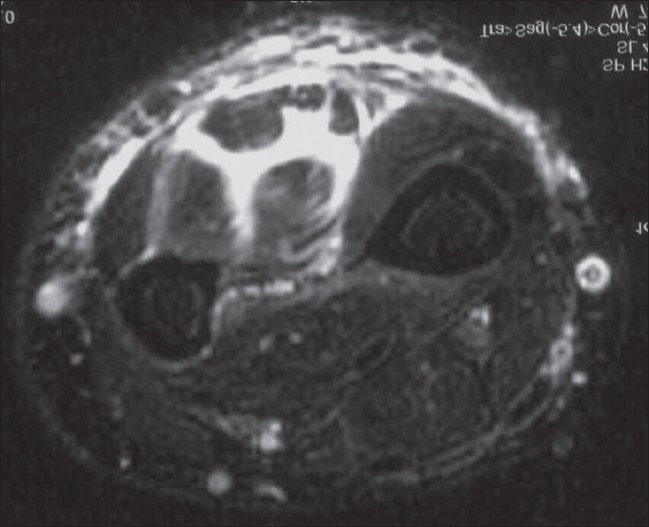
Tuberculous myositis of the forearm. Axial, fat-suppressed, T2W MRI shows high signal intensity within the muscle bellies as well as between the various flexor muscles. There is also increased T2 signal within the adjacent subcutaneous tissue

**Figure 17:**
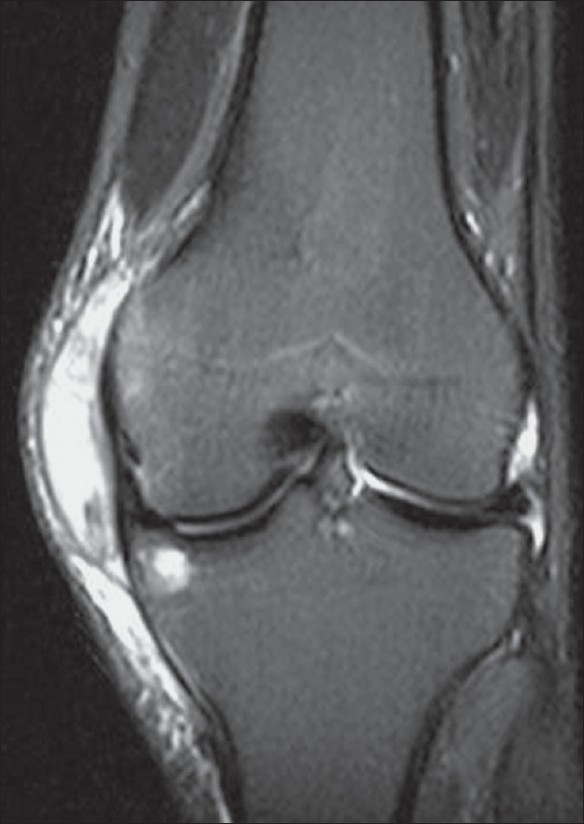
Soft tissue tuberculosis along the medial aspect of the left knee. There is associated osteomyelitis. Coronal, fat-suppressed, T2W MRI shows a high-signal-intensity soft tissue abscess, with adjacent bone marrow edema involving the medial condyle and a T2 hyperintense focus in the medial proximal tibia, due to the associated osteomyelitis

*MRI*: MRI is the preferred imaging method to assess isolated pyomyositis. The lesion is of low signal intensity on T1W images and of high signal intensity on T2W images [Figure 16]. Abscess formation is the rule in all cases of pyomyositis. The peripheral wall of the abscess shows a subtle hyperintensity on T1W images and hypointensity on T2W images. This finding is related to oxygen free radicals and iron within macrophages in the wall of the abscess.[37] After gadolinium contrast injection, peripheral rim enhancement in this abscess wall is observed. Associated cellulitis [Figure 16] and osteoarticular involvement [Figure 17] may also be present.[36] Clinically, the patient presents with a palpable mass that has been present for a long duration, which may mimic a malignant soft tissue mass.[37]

## Conclusion

Extra-axial musculoskeletal TB may involve a wide variety of tissues, including the joints, bones, muscles, tendon sheaths, or synovial bursae, or a combination of these. Imaging studies such as plain radiography, USG, CT scan and, especially, MRI are valuable tools for the diagnosis and accurate evaluation of extraspinal musculoskeletal TB. USG allows a quick evaluation of soft tissue masses, abscesses, joint effusions, and the degree and extent of tendon and tendon sheath involvement. CT scan may be helpful for the detection of osseous or joint involvement, the presence or absence of periosteal reaction and soft tissue calcifications, sclerosis, and soft tissue abscesses. USG and CT scan are particularly useful for guiding fine needle aspiration or biopsy to provide material for histopathological examination, PCR-based assay for mycobacterial DNA, and culture. MRI is the preferred technique to demonstrate early bone marrow changes in tuberculous osteomyelitis and arthritis, joint effusion, and cartilage destruction.

Unfortunately, extraspinal musculoskeletal TB, produces no pathognomonic imaging signs and, in the advanced stages, mimics other disease processes. Therefore, appropriate laboratory tests are mandatory to confirm the diagnosis.
